# Anomaly Detection in Wind Turbines: Persistence-Based Alarm Confirmation for False-Alarm Mitigation and Detection-Latency Trade-Offs

**DOI:** 10.3390/s26123896

**Published:** 2026-06-19

**Authors:** Welker Facchini Nogueira, Miguel Angelo de Carvalho Michalski, Arthur Henrique de Andrade Melani, Luiz David Ricarte de Souza Custodio, Demetrio Cornilios Zachariadis, Gilberto Francisco Martha de Souza

**Affiliations:** 1 Department of Mechatronics and Mechanical Systems Engineering, Polytechnic School, University of Sao Paulo, Av. Prof. Mello Moraes 2231, Cidade Universitária, Sao Paulo 05508-030, SP, Brazil; welkerfacchini@usp.br (W.F.N.); michalski@usp.br (M.A.d.C.M.); melani@usp.br (A.H.d.A.M.); 2 Department of Mechanical Engineering, Polytechnic School, University of Sao Paulo, Av. Prof. Mello Moraes 2231, Cidade Universitaria, Sao Paulo 05508-030, SP, Brazil; david_ricarte@usp.br (L.D.R.d.S.C.); dczachar@usp.br (D.C.Z.)

**Keywords:** wind turbines, anomaly detection detection, condition monitoring, decision policies, false alarm risk and trust control

## Abstract

Anomaly detection models trained exclusively on healthy data are widely used in wind turbine condition monitoring because failure data are scarce, heterogeneous, and often unavailable. However, these models produce anomaly indicators that are sensitive not only to fault-related degradation but also to normal operational variability, transient disturbances, and changes in loading conditions. As a result, the practical behavior of an alarm system depends not only on the anomaly detection model but also on the decision rule used to activate and maintain alarm states. This study presents a decision-oriented evaluation of persistence-based alarm confirmation in wind turbine anomaly detection. Four representative techniques are analyzed within a unified framework: Isolation Forest, One-Class Support Vector Machine, Referenced Moving Window Principal Component Analysis using Q-statistic and percentage component weight indicators, and Autoencoder-based reconstruction error. The evaluation combines controlled OpenFAST simulations of rotor unbalance under different severity and noise conditions with an industrial SCADA case study involving a documented main bearing fault. Results show that temporal persistence strongly shapes alarm outcomes across methods and datasets. Low persistence values favor early detection but promote alarms from isolated threshold exceedances, whereas moderate persistence substantially reduces false positives while preserving detection capability in severe and well-observable faults. Excessive persistence increases detection latency and missed detections, particularly for weak, intermittent, or slowly evolving fault signatures. These findings indicate that persistence-based alarm confirmation should be treated as an explicit decision-level configuration variable, rather than as a fixed post-processing or alarm-state heuristic, when designing anomaly detection systems for wind turbine condition monitoring.

## 1. Introduction

The global transition to renewable energy has placed wind power at the center of decarbonization strategies due to its expansion potential, competitive costs, and low life-cycle emissions [1]. Ensuring reliable operation over the turbine lifetime is therefore critical, as unexpected component degradation directly increases downtime, maintenance costs, and operational risk [2]. Reliability studies consistently show that failures affecting major components, such as gearboxes, generators, and blades, have a disproportionate impact on availability and repair effort, particularly when degradation is not detected at early stages. These effects become increasingly relevant in aging fleets and directly influence maintenance planning, life extension, and asset management decisions [3,4].

Reliability-Centered Maintenance (RCM) and Prognostics and Health Management (PHM) frameworks address these challenges by shifting maintenance strategies from reactive to predictive regimes [5]. Although risk-based perspectives are well established in maintenance planning and asset management [6,7], risk is still predominantly treated at the planning level. Comparatively little attention is given to the decision uncertainty introduced by condition monitoring systems themselves, creating a gap between algorithmic developments in PHM and their translation into reliable and trusted operational decision support [8].

A central challenge in wind turbine fault and anomaly detection lies in the scarcity and heterogeneity of high-quality failure data. Industrial datasets are often incomplete or inaccessible due to confidentiality constraints, motivating the widespread use of simulation environments such as Bladed and OpenFAST to generate controlled fault scenarios [9,10,11]. Even when real data are available, identifying truly healthy operating periods and accurately defining fault onset remain nontrivial tasks [12,13,14]. The absence of open, standardized datasets further limits reproducibility and comparability across studies [15,16,17].

Within this context, anomaly detection techniques trained exclusively on healthy data have become widely adopted in wind turbine monitoring. Methods based on Isolation Forest (IF), One-Class Support Vector Machine (OC-SVM), Principal Component Analysis (PCA), and Autoencoders (AEs) learn representations of nominal behavior and flag deviations as anomalies [18,19,20,21,22,23]. By construction, these detectors are highly sensitive not only to fault-related changes but also to normal operational variability driven by environmental conditions, control actions, and transient load changes. While such sensitivity supports early anomaly awareness, it also increases exposure to false alarms when decisions are triggered by isolated threshold crossings.

Excessive false alarms are a recognized driver of alarm fatigue and operator distrust, often leading alerts to be ignored [24]. Conversely, suppressing alarms through reduced sensitivity can delay detection or result in missed faults, increasing operational risk, particularly for slowly evolving or weakly observable degradation mechanisms. This trade-off reflects fundamental limitations in fault observability and operating conditions rather than deficiencies in algorithm design and cannot be fully resolved at the model level alone. Instead, it emerges at the decision level, where model-generated anomaly evidence must be translated into actionable maintenance signals. Despite the widespread use of persistence-based confirmation rules in practice, their impact on false alarms, missed detections, and detection timing is rarely analyzed systematically across different detection paradigms.

Against this background, the present study adopts a decision-oriented perspective on anomaly detection for wind turbines by evaluating how persistence-based alarm confirmation policies shape the operational behavior of representative detection families within a unified experimental framework. Four widely used techniques are evaluated: IF; OC-SVM; Referenced Moving Window PCA (RMWPCA) [25,26] using two complementary indicators, the percentage component weight (pcw) and the Q-statistic; and an AE. The analysis is conducted using both controlled OpenFAST simulations and real Supervisory Control and Data Acquisition (SCADA) data from an industrial wind turbine operated by EDP.

By jointly analyzing detection latency, false-alarm exposure, and missed-detection risk across simulated and industrial case studies, this work provides practical insights into the configuration of anomaly detection systems for real-world wind turbine monitoring. Unlike benchmarking studies that compare detection models under fixed alarm criteria, the present work treats alarm confirmation as an explicit variable of analysis.

The contributions of this work are threefold: (i) a unified evaluation of representative anomaly detection techniques across simulated and industrial datasets; (ii) a systematic analysis of temporal persistence as an alarm confirmation policy; and (iii) an operational assessment of false-alarm exposure, missed detections, and detection latency. Together, these contributions provide a practical basis for configuring alarm confirmation rules in wind turbine condition monitoring systems.

The selected techniques are not intended to constitute an exhaustive benchmark of all recent wind turbine fault-detection architectures. Rather, they represent distinct and widely used anomaly-indicator families (partition-based, margin-based, projection-based, and reconstruction-based methods) through which the effect of persistence-based alarm confirmation can be analyzed under a common decision-oriented framework.

The remainder of this paper is organized as follows. Section 2 reviews anomaly detection methods for wind turbines and frames the associated uncertainty. Section 3 presents the methodological framework, including Health Indicator (HI) construction, threshold selection, and temporal persistence rules. Section 4 reports the results obtained from simulated and industrial case studies, followed by a discussion. Section 5 concludes the paper and outlines directions for future research.

## 2. Theoretical Background

Anomaly detection techniques aim to characterize nominal system behavior and quantify deviations from this reference as indicators of abnormality, without relying on labeled failure data [26]. This paradigm is particularly suited to wind turbine condition monitoring, where SCADA data are largely unlabeled and documented fault events are scarce, heterogeneous, or incomplete [18,27,28]. Rather than directly identifying fault types, anomaly detection produces continuous indicators that reflect the degree of deviation from normal operation.

In practical monitoring systems, however, these continuous indicators do not constitute decisions by themselves. Translating anomaly evidence into actionable fault detection requires decision rules capable of handling operational variability, noise, and transient disturbances. The need for temporal consistency and alarm validation has long been recognized in fault detection and alarm management literature [29,30,31]. In wind turbine applications, this requirement is commonly addressed through accumulation mechanisms, window-based aggregation, or sustained anomaly criteria, either embedded within detection methods [28] or applied as post-processing to anomaly scores [19,27].

Despite their widespread use, such temporal mechanisms are rarely treated as explicit decision-level design variables. Instead, they are often introduced heuristically or implicitly within specific detection architectures, with limited analysis of their isolated impact on false alarms, missed detections, or detection timing. As emphasized in recent PHM perspectives, this contributes to a persistent gap between algorithmic developments and their translation into reliable operational alarm support for industrial practice [8]. This issue is particularly pronounced in unsupervised and semi-supervised settings, where high sensitivity to deviations may generate unstable alarms, while overly conservative confirmation rules may suppress weak but physically meaningful fault signatures [19,28].

In this work, risk is addressed from an operational perspective, focusing on the observable consequences of decision policies, namely false alarms, missed detections, and delayed interventions, rather than as an abstract probabilistic construct. From this standpoint, the performance of an anomaly detection system depends not only on the detection algorithm itself, but critically on how anomaly indicators are interpreted over time. Temporal persistence rules, which require deviations to be sustained before confirming an alarm, play a central role in balancing early detection, alarm stability, and detection delay. Understanding how different detection mechanisms interact with such decision policies is therefore essential for the reliable deployment of anomaly detection systems in operational wind turbine monitoring.

Although anomaly detection methods share the common objective of learning nominal behavior and quantifying deviations [18,19,20,21,22,23], different algorithmic families operationalize this concept through distinct mechanisms, leading to different sensitivities to noise, operating-condition changes, and fault evolution. In this study, four representative anomaly detection families are considered: partition-based, margin-based, projection-based, and reconstruction-based methods. These families provide a broad methodological basis for analyzing how different anomaly indicators interact with persistence-based alarm confirmation policies under a unified decision-oriented framework.

Partition-based methods, such as IF, identify anomalies by recursively partitioning the feature space using random splits [32]. Observations that are isolated with fewer partitions are considered more anomalous, reflecting their rarity within the data distribution. IF is distribution-free, scalable, and effective in high-dimensional settings, but its sensitivity to rare or extreme observations can lead to fragmented anomaly indications under strong operational variability. In wind turbine applications, IF has demonstrated early detection capability for gearbox, pitch, and bearing faults, while also exhibiting susceptibility to false alarms in highly variable conditions [18,19,22,33].

Margin-based methods, exemplified by the OC-SVM, estimate the support of nominal data by constructing a decision boundary that encloses most healthy observations [34]. Kernel functions allow OC-SVM to capture nonlinear relationships in SCADA data [35], while hyperparameters control the trade-off between sensitivity and boundary smoothness. In practice, OC-SVM often produces smoother anomaly indicators than partition-based methods but may struggle with slowly evolving degradation or subtle drifts [13,18].

Projection-based methods, such as RMWPCA, detect anomalies by monitoring changes in a reduced-dimensional representation relative to a healthy reference subspace [26,36]. By anchoring the projection to a fixed baseline while updating the observation window, RMWPCA aims to balance adaptability and stability. Anomalies may appear as increases in residual variance or as shifts in variance distribution across principal components, enabling sensitivity to correlation changes and gradual structural deviations. While PCA-based monitoring is well established in industrial applications, the use of RMWPCA in wind turbine monitoring remains limited, motivating its inclusion here.

Reconstruction-based methods, most notably AEs, learn compressed latent representations of nominal data and quantify anomalies through reconstruction error [37,38,39]. When trained exclusively on healthy data, AEs capture nonlinear manifolds describing normal operation, with deviations leading to increased reconstruction loss. In wind turbine monitoring, AEs have shown strong performance for gearbox and bearing faults and support extensions such as denoising or temporal modeling [12,23,40]. Their main challenges relate to architectural design, hyperparameter tuning, and interpretability.

Although these detection families differ substantially in their internal mechanisms, they all produce time series of anomaly indicators rather than discrete fault decisions. Consequently, their operational relevance depends critically on how these indicators are transformed into alarms. Temporal persistence provides a systematic means of filtering transient effects and operational variability by requiring anomalies to persist over multiple observations before confirming an alarm [19,27,28,29,30].

From this perspective, false alarms are largely driven by data characteristics and operating context, while missed or delayed detections represent direct operational risk. The interaction between anomaly indicators and persistence-based alarm confirmation policies, therefore, plays a central role in shaping false-alarm exposure, missed detections, and detection latency in condition monitoring systems.

## 3. Methodology

**Figure 1:**
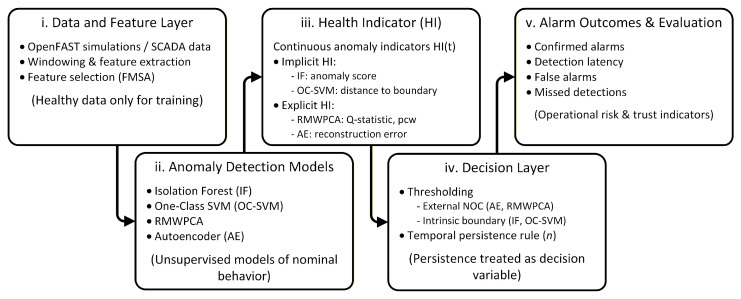
Decision-oriented anomaly detection framework.

The proposed methodology adopts a decision-oriented perspective on anomaly detection for wind turbine condition monitoring by separating anomaly modeling from alarm confirmation. The framework standardizes data representation, HI construction, thresholding, and alarm confirmation rules to enable a consistent comparison across detection families. The workflow, illustrated in Figure 1, is organized into five layers: (i) Data and Feature Layer; (ii) Anomaly Detection Models; (iii) Health Indicator (HI) Layer; (iv) Decision Layer; and (v) Alarm Outcomes and Evaluation.

### 3.1. Data and Feature Layer

This layer defines the data sources, feature construction procedures, and the canonical data partitioning strategy adopted to ensure consistency, reproducibility, and a strict separation between learning and evaluation stages.

Two data domains are considered and processed under the same methodological logic: simulated data and real operational data. The simulated domain consists of time series generated with OpenFAST, including triaxial nacelle vibration signals and operational and aerodynamic variables related to wind conditions, rotational speed, torque, and power [9,10,11]. The real domain comprises 10 min SCADA aggregates obtained from the EDP Open Data platform for industrial wind turbines with documented failures [7,23].

For the simulated datasets, raw signals were segmented into non-overlapping 30 s windows, which define the feature extraction horizon but do not correspond to the decision or alarm confirmation horizon. Each window yields a single feature vector representing the system state over that interval. Vibration signals measured along three orthogonal directions (axial, vertical radial, and horizontal radial) were processed in the time and frequency domains. Time-domain features included RMS and zero-to-peak values, while the amplitude at the first harmonic of the rotor rotational frequency (1×) was extracted in the frequency domain to capture unbalance-related effects. For the remaining operational and aerodynamic variables, mean, RMS, zero-to-peak, and standard deviation were computed within each window.

**Figure 2:**
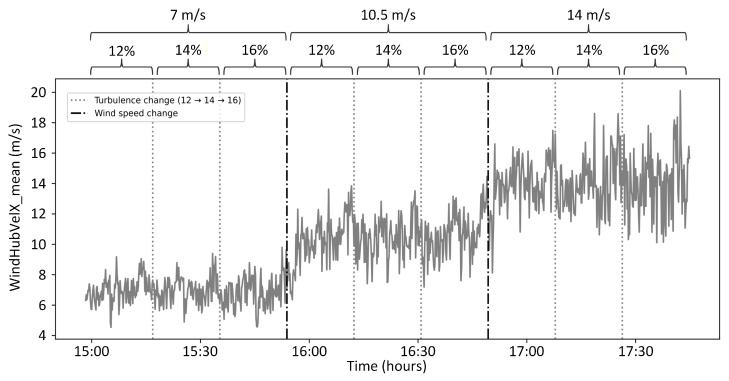
Segmentation of the simulated faulty interval into nine blocks, with step changes marking wind speed (dash–dotted) and turbulence (dotted).

Faulty intervals in the simulated datasets were structured to explicitly control both fault severity and operating conditions. Each time series contained a baseline unbalance level before the fault onset time ($t_{fault}$), followed by an increased unbalance magnitude equal to three (3×) or five (5×) times the baseline value. These severity levels were defined as discrete experimental conditions to control fault observability and facilitate comparison across operating regimes. To ensure comparability across operating regimes, the faulty interval was subdivided into nine operating blocks combining three wind speeds (7, 10.5, and 14 m/s) with three turbulence levels (12%, 14%, and 16%). This design produces stepwise changes in mechanical loading, such that unbalance manifestations emerge primarily as operating-condition-dependent deviations rather than smooth temporal ramps. Figure 2 illustrates this block structure using the mean hub-height wind speed *(WindHubVelX_mean)* as a reference variable.

The complete set of features retained for the simulated unbalance analysis is summarized in Table 1.

To assess robustness under realistic measurement conditions, additive Gaussian white noise proportional to 10% of the RMS value of each raw signal was introduced in selected simulation scenarios. This noise injection acts as a controlled stress test without altering the underlying fault mechanisms.

For the SCADA dataset, no additional windowing or aggregation was required, as measurements are already provided as 10 min aggregates. Each SCADA record, therefore, directly represents a feature vector associated with a specific operating condition. Despite differences in temporal resolution and data origin, both simulated and real datasets are mapped to the same feature-based representation prior to anomaly detection.

The SCADA data correspond to turbine T07 from the EDP Open Data platform, for which a documented main bearing fault is available. Data were collected from an offshore wind farm located in the Gulf of Guinea, West Africa, covering the period from January 2016 to December 2017. The variables selected for this turbine, listed in Table 2, were chosen based on a Failure Mode and Symptoms Analysis (FMSA) [23], extended to preserve the causal chain between failure mechanisms, observable symptoms, and monitoring signals in line with Failure Mode and Observability Analysis (FMOA) principles [26,41]. Accordingly, the selected features focus on thermal and load-related variables physically associated with bearing degradation.

All processed data were organized using a canonical partitioning strategy designed for anomaly detection. Five mutually exclusive subsets were defined: training, testing, threshold, healthy evaluation, and fault evaluation. Training, testing, and threshold subsets contain exclusively healthy observations and are used for model learning, validation, and threshold definition, respectively, while fault data are reserved exclusively for performance assessment.

Fault evaluation subsets were defined first based on domain-specific criteria. In the simulated datasets, fault samples correspond to controlled unbalance conditions at 3× and 5× the baseline level, with an equal number of healthy samples selected to form balanced evaluation sets. In the SCADA dataset, fault periods were defined using documented shutdown events, following established practice in wind turbine condition monitoring studies [7,23]. Specifically, the 60 days preceding the shutdown were labeled as faulty, while the 60 days before this interval were labeled as healthy and used exclusively for evaluation. This 60-day pre-shutdown interval should be interpreted as an operational fault-evaluation horizon rather than as an exact degradation-onset label. Because the actual physical onset is not available from the event log, this conservative horizon helps avoid excluding valid early alarms from the evaluation, although it may reduce recall by including weakly degraded or nearly healthy samples at the beginning of the fault window.

All remaining healthy data were subdivided into threshold, training, and testing subsets. One-third of the healthy observations were reserved for threshold estimation, while the remaining two-thirds were split into training and testing sets using a 60/40 ratio. Although sample selection was random, temporal order was preserved within each subset. This partitioning strategy was applied consistently to both simulated and real datasets, ensuring strict separation between learning and evaluation stages and avoiding ad hoc or data-dependent procedures.

### 3.2. Anomaly Detection Models

This layer describes the anomaly detection models used to learn nominal system behavior from healthy data. At this stage, the focus is restricted to representation learning and anomaly scoring, without introducing decision thresholds, persistence rules, or alarm logic.

All models follow the same learning protocol defined in Section 3.1 and are trained exclusively on healthy observations, ensuring that detected deviations correspond to departures from nominal behavior rather than supervised class discrimination. Before training, all input variables were standardized using z-score normalization based solely on training-set statistics to avoid scale dominance and data leakage. For the SCADA dataset, samples with active power below 100 kW were discarded to exclude startup, shutdown, and non-operational conditions.

Four representative anomaly detection techniques were considered, each corresponding to a distinct methodological family widely adopted in wind turbine condition monitoring: IF, OC-SVM, RMWPCA, and AE.

IF is a partition-based method that identifies anomalies through random recursive partitioning of the feature space [32]. Samples that are isolated with fewer splits are assigned higher anomaly scores, reflecting their rarity relative to nominal data. IF is distribution-free, scalable, and well-suited to heterogeneous feature spaces typical of SCADA and simulation-based monitoring. Hyperparameters were optimized via 10-fold cross-validated grid search on healthy training data, exploring contamination ∈ {0.05, 0.07, 0.10}, number of trees ∈ {128, 256, 512}, fraction of training samples per tree ∈ {0.50, 0.75, 1.0}, and fraction of features per split ∈ {0.75, 1.0}. The configuration yielding the highest mean validation accuracy was retained.

OC-SVM is a margin-based approach that estimates the support of the nominal data distribution by constructing a decision boundary enclosing most healthy observations [34]. Using a radial basis function kernel, OC-SVM captures nonlinear relationships among monitored variables, with anomaly scores derived from the distance to the learned boundary. Hyperparameters controlling boundary smoothness, i.e., ν ∈ [0.01, 0.10] (step 0.01), and the expected fraction of outliers, i.e., γ ∈ {$2^{-5},2^{-4},\cdots ,2^{1},2^{2}$}, were optimized via cross-validation on healthy data through 10-fold grid search.

RMWPCA is a projection-based method that extends classical PCA by combining a fixed reference subspace representing healthy operation with a moving observation window [25,26]. This formulation anchors the projection to nominal behavior while allowing adaptation to operational variability. A non-centered formulation was adopted [42,43], enabling sensitivity to both correlation changes and absolute magnitude shifts, which are relevant for physical degradation processes. The reference subspace was constructed exclusively from healthy training data, and the window length was selected based on expected fault evolution rates [41].

AE is a reconstruction-based neural network trained to compress input data into a low-dimensional latent representation and reconstruct it at the output [39]. When trained exclusively on healthy data, the AE learns a manifold representing nominal operation, and deviations from this manifold result in increased reconstruction error. A symmetric feedforward architecture with tanh activation functions was adopted, following established practice in wind turbine monitoring [23]. Models were trained for a fixed number of epochs on standardized inputs, relying on architectural constraints rather *than* explicit regularization to limit overfitting [44,45].

The final model configurations adopted for the simulated scenarios and the SCADA case are summarized in Table 3 and Table 4, respectively.

These configurations correspond to the hyperparameter sets selected during training and validation using healthy data only. Reported accuracy values are provided solely as internal references for model selection and are not intended as comparative performance results, which are assessed later at the decision and alarm outcome levels.

At the output of this layer, each model produces a continuous anomaly-related signal for each observation. These signals are not interpreted as alarms at this stage; instead, they constitute the raw inputs to the subsequent HI layer, where anomaly evidence is formalized into a unified representation suitable for decision-level analysis.

### 3.3. Health Indicator (HI): Explicit and Implicit Representations

The HI layer provides a unified abstraction through which the outputs of heterogeneous anomaly detection models are interpreted and compared at the decision level. Although the internal mechanisms of the considered models differ substantially, all of them produce continuous signals that quantify deviations from nominal system behavior. In this work, such signals are formalized as a HI [46], denoted as *HI*(*t*), and defined over the sequence of observations.

Introducing the HI explicitly shifts the analysis from algorithm-specific outputs to a common representation that supports consistent thresholding, persistence analysis, and decision-level evaluation. This abstraction is essential because alarm decisions ultimately depend on the temporal behavior of a scalar (or low-dimensional) indicator, regardless of how deviations are computed internally by each model.

Two classes of HI representations are distinguished: implicit and explicit indicators. For partition-based (IF) and margin-based (OC-SVM) methods, the HI is implicit and directly derived from the native model output, whereas for projection-based (RMWPCA) and reconstruction-based (AE) methods, the HI is explicitly defined as an interpretable quantity derived from the model output (i.e., Q-statistic and pcw for RMWPCA, and reconstruction error for AE). Table 5 summarizes the raw outputs of each method and the corresponding HI definitions adopted in this study.

For IF, the HI corresponds to the anomaly score derived from the average path length required to isolate a sample within the ensemble of random trees. Shorter path lengths indicate observations that are easier to isolate and therefore more anomalous, providing a continuous measure of deviation based on data rarity.

For OC-SVM, the HI is defined as the signed distance to the learned decision boundary, with larger values indicating samples lying further outside the nominal data support. In both cases, the HI is produced intrinsically by the detection model and does not require external definition. However, these implicit indicators are model-dependent and may be sensitive to local data density, noise, or boundary effects, which can affect their temporal stability.

In the case of RMWPCA, two complementary indicators are considered. The Q-statistic [47,48] quantifies the residual variance not captured by the healthy reference subspace and is primarily sensitive to magnitude-based deviations. The pcw [25] indicator tracks how variance is redistributed among the leading principal components relative to the healthy baseline, enabling sensitivity to correlation changes even when overall residual variance remains within nominal bounds. Together, these indicators allow RMWPCA to capture both energy-related and structural degradation patterns.

For the AE, the HI is defined as the Mean Squared Error (MSE) between the input feature vector and its reconstruction. When trained exclusively on healthy data, the AE learns a manifold representing nominal operation, and deviations from this manifold result in increased reconstruction error. The MSE therefore provides a direct and interpretable measure of deviation magnitude.

By expressing all anomaly detection outputs in terms of a unified *HI(t)*, this layer decouples anomaly representation from decision logic. This decoupling is central to the decision-oriented perspective adopted in this work, as it allows the subsequent Decision Layer to operate identically across models, independent of whether the underlying HI is implicit or explicit. Differences in *HI* construction nonetheless have practical implications for interpretability, temporal smoothness, and sensitivity to operational variability, which directly influence how thresholding and persistence rules shape alarm outcomes, as analyzed in the following section.

### 3.4. Decision Layer

The Decision Layer formalizes how continuous *HIs* produced by the anomaly detection models are translated into discrete alarm events. This layer is deliberately decoupled from the internal structure of the detection algorithms, allowing decision policies to be analyzed independently of the anomaly representation mechanism.

#### 3.4.1. Threshold Definition

Two thresholding strategies are adopted, depending on the anomaly detection model and the associated HI formulation. For IF and OC-SVM, binary anomaly labels are obtained from the intrinsic decision functions defined by the trained models. The corresponding decision boundaries are determined during model fitting from healthy training data and the selected hyperparameters, rather than from fault data or from an externally estimated HI threshold.

For projection-based (RMWPCA) and reconstruction-based (AE) methods, thresholds are defined externally using Normal Operating Condition (NOC) data [49]. In these cases, anomaly detection relies on explicitly constructed HIs (Q-statistic and pcw for RMWPCA; reconstruction error for AE), whose distributions under healthy operation are estimated non-parametrically. Thresholds are set using upper quantiles of the NOC distributions, ensuring that alarm triggering reflects statistically significant departures from nominal behavior rather than model-specific score scaling. This approach follows established practice in PCA-based monitoring and autoencoder-based fault detection, particularly in the absence of labeled fault data.

In all cases, threshold or decision-boundary definition relies exclusively on healthy observations and does not involve fault data, preserving the unsupervised nature of the detection problem.

#### 3.4.2. Temporal Persistence as a Decision Variable

Threshold exceedance alone is insufficient to confirm an alarm. To enforce temporal consistency and suppress spurious detections caused by noise, transient disturbances, or short-lived operational changes, a temporal persistence rule is applied uniformly across all techniques.

Let z(t) denote the binary anomaly indication at time index *t*, obtained after thresholding the corresponding health indicator or applying the model-native decision boundary. Thus, z(t)=1 indicates an anomalous observation, and z(t)=0 indicates a normal observation. The persistent alarm state is denoted by a(t).

Persistence is implemented as a causal flip-flop rule with symmetric activation and deactivation criteria. An alarm is activated only after *n* consecutive anomalous indications, i.e., z(t)=1 for *n* consecutive observations, and remains active until *n* consecutive normal indications are observed. Therefore, the same persistence length *n* is used both to confirm alarm activation and to confirm return to the normal state.

This rule represents a conservative alarm-confirmation policy with memory: any interruption in the sequence of anomalous indications resets the activation count, while an active alarm is not cleared by isolated normal observations. More tolerant alternatives, such as *m*-out-of-*n* sliding-window rules, could reduce sensitivity to isolated drops below threshold but would introduce an additional decision parameter beyond the scope of the present analysis.

Under this implementation, the detection index $index_{det}$ is defined as the first sample at which the persistent alarm state becomes active, that is, the observation at which the *n*-consecutive-anomaly condition is first satisfied. Consequently, $index_{det}$ represents the operational alarm-confirmation point, not the first raw threshold exceedance within the anomalous sequence.

The persistence parameter *n* operates strictly at the decision level and is independent of the feature extraction procedure.

It therefore functions as a consistency filter applied to the binary anomaly sequence and persistent alarm state, rather than as a signal-processing operation. However, although *n* is defined in number of consecutive observations, its operational meaning also depends on the sampling or aggregation interval Δt. Persistence can therefore be interpreted in physical time as a confirmation duration $T_{p}=n\Delta t$, which is particularly important when comparing datasets with different temporal resolutions, such as the 30 s simulation windows and the 10 min SCADA records considered in this study.

Varying *n* explicitly controls the trade-off between early detection and alarm stability. Low persistence values favor rapid detection but increase exposure to false alarms, whereas higher persistence values suppress spurious alarms at the cost of increased detection latency and potential missed detections, particularly for weak or slowly evolving faults.

#### 3.4.3. Operational Interpretation of Risk

Within this framework, risk is interpreted from an operational perspective, focusing on the observable consequences of decision policies, i.e., false alarms, missed detections, and delayed interventions, rather than through abstract probabilistic or economic formulations.

From this standpoint, false alarms primarily arise from the interaction between data variability, operating context, and decision policy, while missed or excessively delayed detections represent direct operational risk. The persistence parameter thus emerges as a central design variable for balancing responsiveness, false-alarm mitigation, and timely detection in condition monitoring systems.

By applying identical thresholding and persistence logic across all anomaly detection families, the proposed methodology isolates the effect of decision policies from algorithmic differences, enabling a fair and explicitly decision-oriented comparison of detection outcomes in the subsequent evaluation stage.

### 3.5. Alarm Outcomes and Evaluation

This final layer evaluates the alarm outcomes resulting from the interaction between anomaly detection models, HIs, and decision policies. Beyond conventional classification performance, the analysis focuses on how detection behavior evolves with temporal persistence and operating conditions, from an operational risk perspective. Evaluation procedures were identical for simulated and real datasets, ensuring direct comparability across anomaly detection families and decision policies.

To explicitly assess the role of decision policies, the persistence length *n* was treated as an experimental variable rather than a fixed design choice. For each technique, alarm outcomes were evaluated over increasing values of *n*, ranging from immediate detection (n=1) to progressively more conservative confirmation rules. This systematic variation enables a direct analysis of how temporal consistency requirements affect false alarms, missed detections, detection latency, and overall alarm stability.

For the simulated datasets, evaluation was conducted under controlled fault scenarios, allowing systematic variation of both operating conditions and fault severity. Two factors were considered: fault severity, modeled as rotor unbalance levels three and five times higher than the baseline reference, and measurement uncertainty, introduced through additive Gaussian white noise proportional to 10% of the RMS value of each signal. These combinations define four simulation conditions, enabling assessment of robustness under both idealized and noisy measurement environments. Performance metrics were computed separately for each condition, isolating the effects of persistence, noise, and fault magnitude.

For the real SCADA dataset, alarm outcomes were compared against operation and maintenance records to verify consistency between detected anomalies and documented degradation periods. The same persistence values and evaluation metrics were applied as in the simulated analysis, allowing assessment of whether trends observed under controlled conditions persist under real operational variability.

Performance assessment combined sample-level classification metrics with time-resolved operational indicators. Classification metrics were retained to support comparability across techniques and were computed using balanced healthy and fault evaluation subsets, including Accuracy, Precision, Recall, F1-score, and the corresponding confusion-matrix counts (TPs, TNs, FPs, FNs). These metrics were computed from the persistent alarm state, rather than directly from raw threshold exceedances, so that classification outcomes reflect the same alarm-confirmation logic used in the time-resolved analysis. The operational interpretation focused on false-alarm exposure, missed detections, and detection latency. To capture detection dynamics, time-resolved indicators included the detection index, $index_{det}$, defined as the first sample at which the persistent alarm state becomes active; the corresponding detection timestamp; and the detection latency, computed as the difference between the detection time, $t_{det}$, and the fault onset time, $t_{fault}$, and reported in both samples and minutes. Thus, $t_{det}$ represents the operational confirmation time associated with the persistence rule, rather than the first raw threshold crossing. Negative latency values indicate early detection. Missed detections were recorded explicitly as cases in which no persistent alarm was confirmed within the fault evaluation window.

From a decision-oriented standpoint, alarm outcomes are interpreted in terms of their operational consequences rather than purely statistical performance. False alarms reflect unnecessary alarm exposure and potential inspection burden, while missed or excessively delayed detections represent direct operational risk. By jointly analyzing confusion-matrix metrics and detection latency as functions of temporal persistence, the proposed methodology enables a transparent and systematic assessment of how anomaly representations and alarm confirmation policies jointly shape the reliability and operational usefulness of wind turbine condition monitoring systems.

## 4. Results and Discussion

This section reports the results obtained from the simulated rotor-unbalance scenarios (Section 4.1) and the real SCADA dataset of turbine T07 (Section 4.2), followed by an integrated discussion (Section 4.3).

The analysis follows the separation between anomaly indication and alarm confirmation adopted in this work. First, the results are examined under the immediate decision setting (n=1), in which alarms are triggered by a single threshold crossing. This stage uses confusion matrices and time-resolved detection plots to characterize the raw response of each method before temporal validation.

Second, temporal persistence is evaluated by analyzing how performance metrics and detection timing change as the persistence length *n* increases from 1 to 20. This step treats persistence as a configurable alarm-confirmation parameter, rather than as an intrinsic property of the detection model. Together, these analyses distinguish model-level anomaly sensitivity from decision-level alarm robustness under different operating conditions and confirmation policies.

### 4.1. Simulated Dataset Results

**Figure 3:**
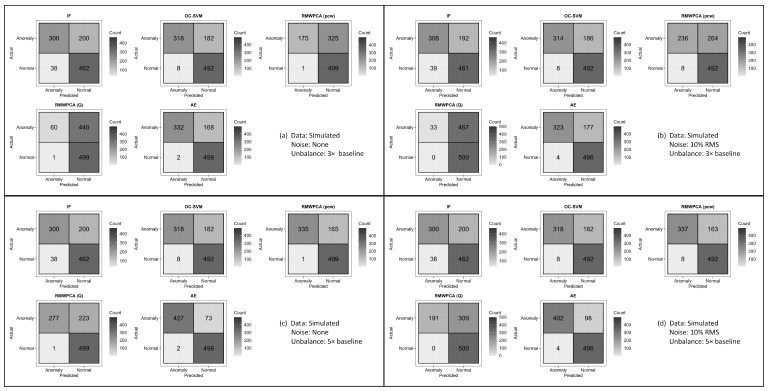
Confusion matrices for simulated unbalance scenarios. Panels: IF, OC-SVM, RMWPCA-Q, RMWPCA-pcw, and AE under (**a**) no noise, 3× baseline; (**b**) 10% RMS noise, 3× baseline; (**c**) no noise, 5× baseline; (**d**) 10% RMS noise, 5× baseline.

**Figure 4:**
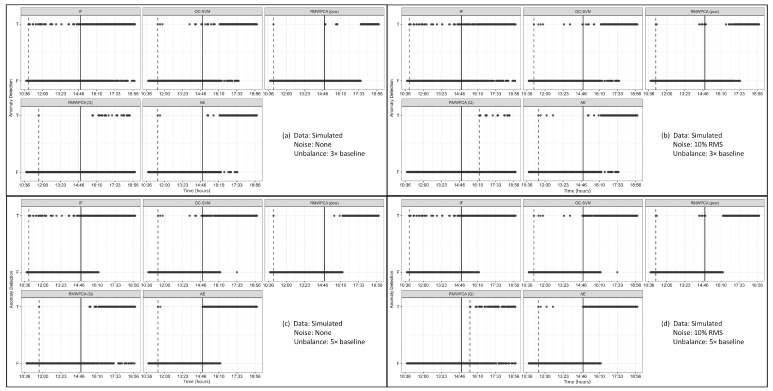
Time-resolved anomaly detections for simulated unbalance scenarios with persistence length n=1. Panels: IF, OC-SVM, RMWPCA-Q, RMWPCA-pcw, and AE under (**a**) no noise, 3× baseline; (**b**) 10% RMS noise, 3× baseline; (**c**) no noise, 5× baseline; (**d**) 10% RMS noise, 5× baseline. Solid vertical line: fault onset $\left(t_{fault}\right)$; dashed line: detection time $\left(t_{det}\right)$ after persistence rule.

This subsection presents the results obtained for the simulated rotor-unbalance scenarios, considering two fault severities (3× and 5× the baseline unbalance) and two measurement conditions (no noise and 10% RMS additive noise), yielding four scenarios in total. Results are first examined under the immediate decision setting (n=1), in which alarms are confirmed upon a single threshold exceedance. Table 6 and Figure 3 summarize the corresponding confusion-matrix outcomes, while Figure 4 provides the associated time-resolved anomaly detections. As persistence is not enforced at this stage, the reported counts depend solely on the prescribed fault onset time $t_{fault}$.

Across all scenarios, the confusion-matrix metrics indicate high precision and stable accuracy for most techniques, suggesting limited exposure to false positives under immediate decision rules. However, these aggregate indicators mask important temporal effects. The time-resolved detections in Figure 4 show that false positives are predominantly associated with isolated threshold exceedances occurring before fault onset or around operating-regime transitions. When decisions are based on single samples, such transient activations are promoted directly to alarms despite lacking temporal consistency.

This explains why techniques such as OC-SVM and AE, which exhibit strong precision and accuracy, may still generate unstable alarm behavior under n=1. Conversely, conservative indicators such as the RMWPCA Q-statistic suppress spurious activations but incur substantial missed detections in low-severity faults. These observations indicate that perceived false-alarm proneness arises primarily from the absence of temporal validation, rather than from globally elevated false-positive rates. Figure 4, therefore, illustrates that good sample-level classification performance does not necessarily translate into reliable alarm behavior at the decision level, motivating the explicit analysis of temporal persistence as an alarm-confirmation parameter.

**Figure 5:**
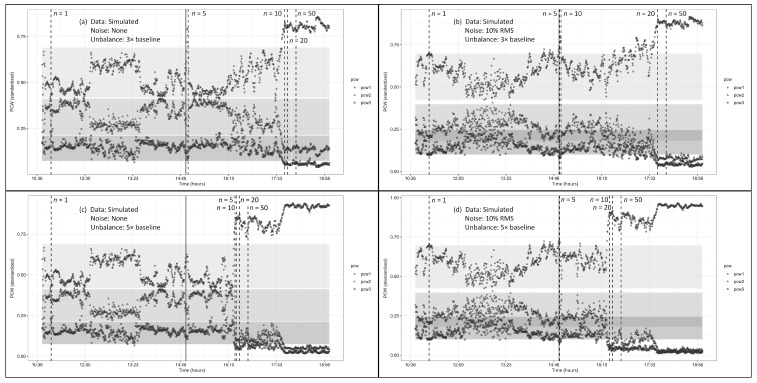
Time-resolved health indicators from RMWPCA-pcw for simulated unbalance scenarios. Panels: (**a**) no noise, 3× baseline; (**b**) 10% RMS noise, 3× baseline; (**c**) no noise, 5× baseline; (**d**) 10% RMS noise, 5× baseline. Shaded regions indicate normality bounds; dashed vertical lines indicate confirmed detections for n∈{1,5,10,20,50}.

**Figure 6:**
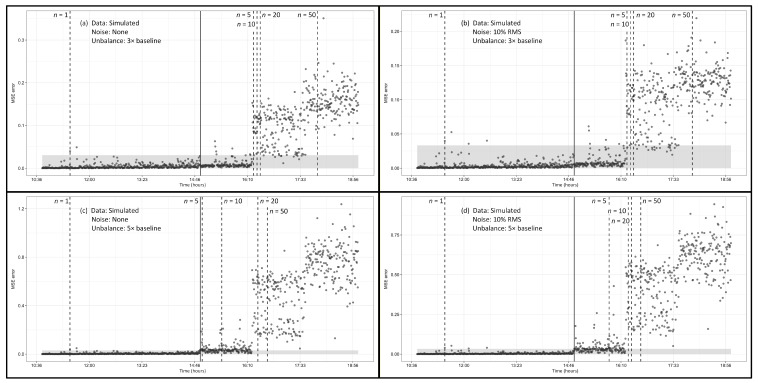
Time-resolved health indicators from AE reconstruction error (MSE) for simulated unbalance scenarios. Panels: (**a**) no noise, 3× baseline; (**b**) 10% RMS noise, 3× baseline; (**c**) no noise, 5× baseline; (**d**) 10% RMS noise, 5× baseline. Shaded regions indicate normality bounds; dashed vertical lines indicate confirmed detections for n∈{1,5,10,20,50}.

The temporal structure of anomaly evidence is further clarified by the explicit health indicators produced by RMWPCA-pcw and AE. As shown in Figure 5 and Figure 6, the pcw and MSE trajectories reveal that threshold crossings cluster within specific operating blocks. Early threshold crossings are confirmed under n=1, whereas only sustained departures remain detectable as persistence increases. This behavior directly links the persistence parameter to detection time $t_{det}$.

The structure of the simulated data primarily explains false negatives observed in low-severity scenarios. As described in Section 3.1, the faulty interval is divided into operating blocks with different wind speeds and turbulence levels. Under low-load conditions, the turbine response to unbalance remains weak, resulting in limited vibration and load deviations. In these regimes, fault signatures may remain below threshold or fail to persist long enough to satisfy stricter alarm-confirmation rules, particularly for magnitude-driven indicators such as the Q-statistic. As operating conditions become more demanding, deviations become stronger and more coherent, enabling sustained threshold exceedances and earlier detection, even at higher persistence values.

**Figure 7:**
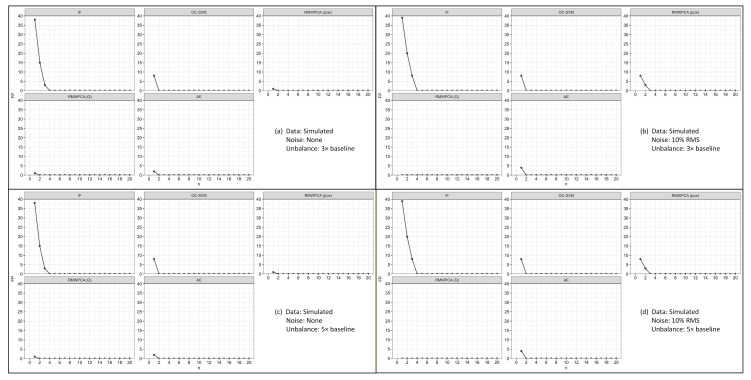
False Positives (FPs) as a function of persistence length *n* for simulated unbalance scenarios. Panels: IF, OC-SVM, RMWPCA-Q, RMWPCA-pcw, and AE under (**a**) no noise, 3× baseline; (**b**) 10% RMS noise, 3× baseline; (**c**) no noise, 5× baseline; (**d**) 10% RMS noise, 5× baseline.

**Figure 8:**
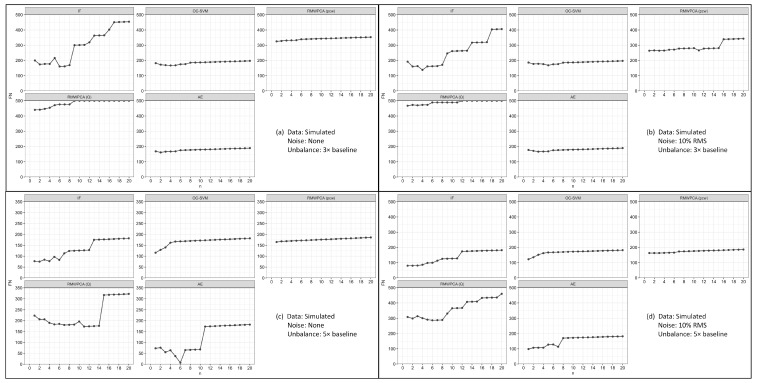
False Negatives (FNs) as a function of persistence length *n* for simulated unbalance scenarios. Panels: IF, OC-SVM, RMWPCA-Q, RMWPCA-pcw, and AE under (**a**) no noise, 3× baseline; (**b**) 10% RMS noise, 3× baseline; (**c**) no noise, 5× baseline; (**d**) 10% RMS noise, 5× baseline.

**Figure 9:**
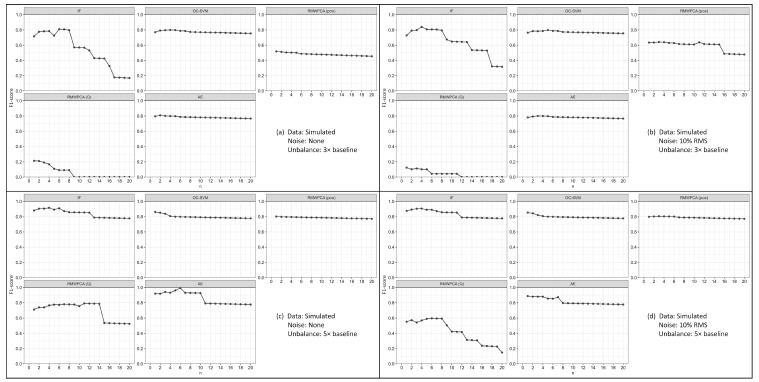
F1-Score as a function of persistence length *n* for simulated unbalance scenarios. Panels: IF, OC-SVM, RMWPCA-Q, RMWPCA-pcw, and AE under (**a**) no noise, 3× baseline; (**b**) 10% RMS noise, 3× baseline; (**c**) no noise, 5× baseline; (**d**) 10% RMS noise, 5× baseline.

**Figure 10:**
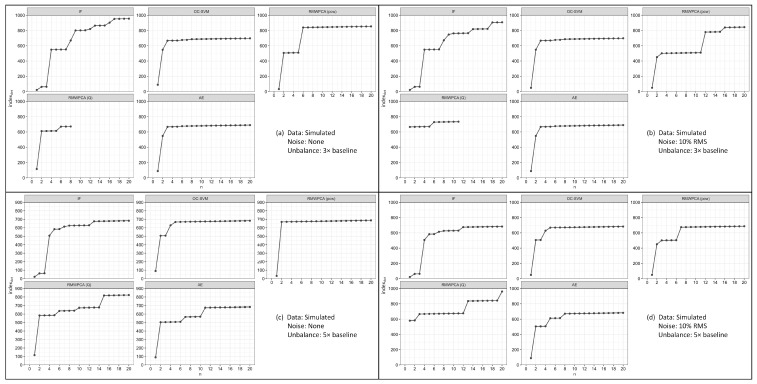
Detection index $\left(index_{det}\right)$ as a function of persistence length *n* for simulated unbalance scenarios. Panels: IF, OC-SVM, RMWPCA-Q, RMWPCA-pcw, and AE under (**a**) no noise, 3× baseline; (**b**) 10% RMS noise, 3× baseline; (**c**) no noise, 5× baseline; (**d**) 10% RMS noise, 5× baseline.

The effect of temporal persistence on alarm outcomes is summarized in Figure 7, Figure 8, Figure 9 and Figure 10, which report False Positives (FPs), False Negatives (FNs), F1-score, and detection index $\left(index_{det}\right)$ as functions of the persistence length *n*. Figure 7 shows that false alarms are rapidly suppressed with modest persistence values, typically for n≤3 to 5 in cases where false positives are present. This confirms that false alarms observed under n=1 arise mainly from isolated threshold crossings rather than sustained detector instability, supporting the interpretation of persistence as a largely model-agnostic mechanism for stabilizing the alarm state.

In contrast, Figure 8 shows that increasing persistence progressively shifts the dominant error mode toward false negatives, particularly in the 3× baseline scenarios. Weak and intermittent fault signatures often fail to remain above threshold long enough to activate or maintain the persistent alarm state, an effect most pronounced for the Q-statistic but observable across all methods. For 5× baseline unbalance, the growth of false negatives with persistence is generally attenuated due to stronger and more coherent physical signatures.

The combined effect of decreasing FPs and increasing FNs is reflected in the F1-score trends shown in Figure 9. For most techniques, the F1-score either peaks at low persistence values or remains highest under low-to-moderate persistence, where false alarms are already strongly reduced, while recall remains sufficiently high. As persistence increases further, the F1-score tends to degrade due to the loss of recall associated with delayed or suppressed alarm states. This confirms that favorable performance does not correspond to maximal persistence, but rather to a decision region balancing early detection and alarm stability.

Finally, Figure 10 shows that the detection index generally increases with persistence, indicating delayed alarm confirmation. The rate of increase varies across techniques and scenarios because $index_{det}$ is defined as the point at which the persistent alarm state first becomes active. AE and OC-SVM preserve relatively early detection in several scenarios, particularly for more severe faults, whereas conservative indicators may exhibit abrupt delays or complete loss of detection at higher persistence values.

Taken together, these results demonstrate that temporal persistence is not merely a noise-filtering mechanism but a dominant alarm-confirmation parameter that reshapes alarm outcomes. While small persistence values are sufficient to suppress spurious alarms without substantially compromising detectability in severe-fault scenarios, overly conservative persistence shifts the main operational burden from false alarms to delayed or missed detections in incipient faults. Consequently, in the simulated cases, alarm behavior is governed more strongly by the chosen alarm-confirmation policy than by the anomaly detection algorithm itself.

### 4.2. SCADA Dataset Results

**Figure 11:**
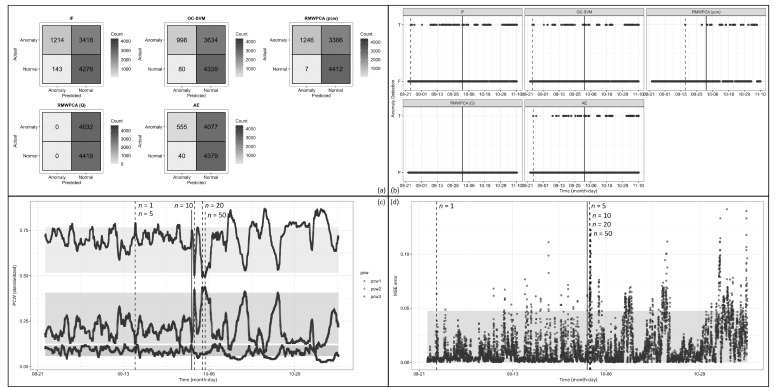
Results for turbine T07 (main bearing fault): (**a**) confusion matrices; (**b**) time-resolved anomaly detections with persistence length n=1; (**c**) health indicators from RMWPCA-pcw; (**d**) reconstruction errors from AE.

Following the simulated analysis, this subsection presents the results obtained for the T07 SCADA dataset, corresponding to a documented main bearing fault. The analysis first examines the immediate decision setting (n=1), in which alarms are confirmed upon single threshold exceedances. Figure 11 summarizes the baseline results, including confusion matrices, time-resolved detections, and representative health indicator trajectories, while Table 7 consolidates the associated performance metrics.

Under the immediate decision rule, all techniques exhibit high precision, ranging from 0.89 to 0.99, indicating limited exposure to false positives in real SCADA data. However, recall is uniformly low across methods, reflecting the gradual progression and weak observability of the bearing fault in the monitored variables.

IF and OC-SVM achieve accuracies of 0.61 and 0.59, respectively, but identify less than one quarter of the faulty samples. RMWPCA-pcw attains the highest accuracy (0.63) and recall (0.27), suggesting improved sensitivity to correlation changes captured by the pcw trajectories. In contrast, the AE shows limited sensitivity (recall 0.12), while the RMWPCA Q-statistic fails to trigger any detection, remaining entirely below threshold throughout the evaluation period.

The time-resolved detections further clarify these results by showing that anomaly activations occur as sparse and intermittent threshold crossings rather than sustained alarm regions. As in the simulated cases, these isolated activations explain the combination of high precision and low recall under n=1 and indicate that thresholding alone is insufficient to support reliable alarm behavior in the presence of slow degradation. The HI trajectories confirm that fault-related deviations become pronounced only during specific operating intervals, remaining close to nominal levels for extended periods.

Overall, these baseline results indicate that conservative indicators such as the Q-statistic exhibit limited sensitivity to gradual bearing degradation, while RMWPCA-pcw and the autoencoder provide complementary but intermittent evidence of abnormal behavior. IF and OC-SVM act as stable baselines, preserving precision but showing reduced sensitivity under slow fault progression. Consistent with the simulated analysis, these findings motivate the explicit evaluation of temporal persistence as an alarm-confirmation parameter.

**Figure 12:**
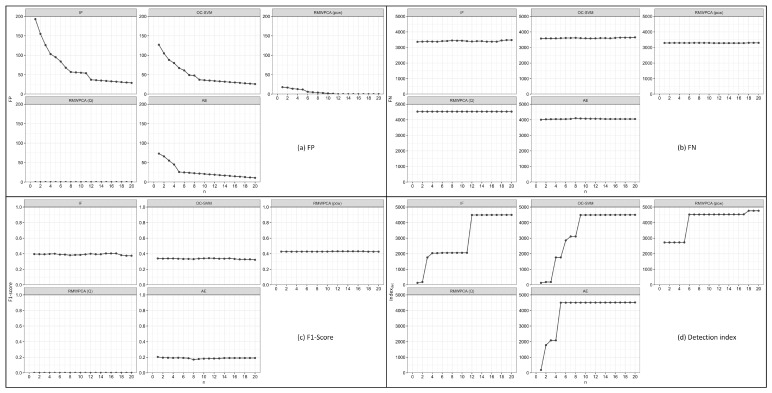
Effect of persistence length *n* on detection outcomes for turbine T07 (main bearing fault). Panels: (**a**) false positives (FPs), (**b**) false negatives (FNs), (**c**) F1-score, and (**d**) detection index $\left(index_{det}\right)$ as functions of *n* for IF, OC-SVM, RMWPCA-pcw, RMWPCA-Q, and AE.

The impact of temporal persistence on alarm outcomes for turbine T07 is summarized in Figure 12, which reports the evolution of false positives, false negatives, F1-score, and detection index as functions of the persistence length n.

As observed in Figure 12, false-alarm exposure is rapidly suppressed with modest persistence values, typically for n≈3 to 5, confirming that false alarms are largely driven by isolated threshold exceedances rather than sustained abnormal behavior.

In contrast, false negatives remain high and tend to increase with persistence, more markedly than in the simulated cases. This reflects the gradual nature of the bearing degradation, whose signatures manifest intermittently and with limited amplitude. As persistence increases, anomaly indicators frequently fail to remain above threshold long enough to activate the persistent alarm state, redistributing alarm outcomes from false-positive exposure toward missed or delayed detections. This effect is particularly evident for conservative indicators, including the Q-statistic, which fails to register detections across all persistence values.

The corresponding F1-score trends show that increasing persistence does not improve performance and, for most techniques, either preserves low-to-moderate F1-score values or leads to degradation as recall decreases. Unlike the simulated high-severity scenarios, no persistence range yields a stable performance plateau, highlighting the difficulty of balancing alarm stability and sensitivity in real SCADA data affected by slow and weak fault progression. Detection index results further confirm this behavior, showing stepwise or nearly flat changes in alarm-confirmation delay with persistence. IF and OC-SVM retain some earlier alarm-confirmation capability under moderate persistence, whereas RMWPCA-pcw and AE exhibit more abrupt detection delays, with alarms concentrated near the end of the fault window.

These differences can be explained by the interaction between the flip-flop persistence rule and the temporal structure of the binary anomaly sequences generated by each method. Since alarm activation requires *n* consecutive anomalous indications, $index_{det}$ is assigned to the observation at which this condition is first satisfied. Therefore, increasing *n* does not necessarily produce a smooth large-scale increase in detection delay. While the same anomalous sequence remains valid, $index_{det}$ changes only gradually within that sequence; if the sequence becomes insufficient, confirmation shifts to a later sustained anomalous episode, producing an abrupt increase. In addition, because the persistent alarm state remains active until *n* consecutive normal indications occur, persistence also affects FPs, FNs, and F1-score by controlling both alarm activation and alarm deactivation. Thus, the patterns observed in Figure 12d reflect the duration, intermittency, and temporal distribution of method-specific anomaly indications under the gradual and weakly observable T07 bearing fault.

Overall, the T07 results demonstrate that temporal persistence remains a dominant alarm-confirmation parameter under real operating conditions, but its effects are amplified by limited fault observability and gradual degradation dynamics. While small persistence values effectively suppress false alarms, increasing persistence maintains or increases the burden of missed detections and may lead to excessively delayed alarms. These findings reinforce that, in practical SCADA-based monitoring, alarm behavior is governed more strongly by the chosen alarm-confirmation policy than by the anomaly detection algorithm itself, and that persistence tuning must explicitly account for fault severity, progression rate, and operational context.

### 4.3. Results Discussion

The combined analysis of the simulated unbalance scenarios and the T07 SCADA dataset consistently shows that alarm behavior in anomaly detection systems is shaped not only by the detection algorithm but predominantly by the alarm-confirmation policy applied to the anomaly indicators. By explicitly separating anomaly indication from alarm confirmation, this study clarifies how temporal persistence shapes persistent alarm states and redistributes detection outcomes across different fault severities, noise levels, and operating conditions.

In the simulated scenarios, baseline results obtained under immediate decision rules (n=1) suggest high precision and stable accuracy across all techniques. However, time-resolved analyses reveal that false alarms are largely driven by isolated threshold exceedances occurring before fault onset or during operating-regime transitions. These transient activations are indistinguishable from sustained fault-related behavior when alarms are triggered on single samples, demonstrating that sample-level classification performance alone is insufficient to ensure reliable alarm behavior.

Introducing temporal persistence fundamentally changes this picture. Across all simulated conditions, modest persistence values (n≈ 3 to 5) are sufficient to suppress false alarms almost entirely, indicating that FPs arise primarily from the absence of temporal validation rather than from persistent detector instability. This persistence range should be interpreted as case-dependent, since the appropriate range of *n* depends on the temporal resolution of the data, the temporal smoothness and intermittency of the health indicator, the fault progression rate, and the observability of the monitored failure mechanism. At the same time, increasing persistence progressively shifts the dominant error mode toward FNs, particularly for low-severity faults. This trade-off reflects the physical structure of the simulated system: under low wind speeds and turbulence, unbalance-induced deviations remain weak and intermittent, becoming observable only when operating conditions amplify mechanical loads. In this context, missed detections are more strongly associated with limited fault observability than with algorithmic shortcomings.

The evolution of the F1-score corroborates this interpretation. Across all simulated cases, F1 peaks at low-to-moderate persistence values, where false alarms are already strongly reduced, while recall remains sufficiently high. As persistence increases further, F1 tends to degrade due to the loss of recall associated with delayed or suppressed detections. These results indicate that favorable alarm behavior emerges in a narrow decision region that balances stability and sensitivity, rather than at maximal conservatism.

Detection timing analyses further confirm that the flip-flop persistence rule acts as a direct control on alarm latency and alarm-state stability. The detection index generally increases with persistence, but at different rates, depending on the anomaly indicator and fault severity. Methods such as AE and OC-SVM preserve earlier detection under moderate persistence, particularly for severe faults, whereas more conservative indicators exhibit abrupt detection delays or complete loss of detection. This behavior reflects the interaction between the persistence rule and the temporal structure of the binary anomaly sequence generated by each model. While the same anomalous sequence remains valid, increasing *n* changes the detection index only gradually within that sequence; if the sequence becomes insufficient, alarm confirmation shifts to a later sustained anomalous episode, producing abrupt delays or missed detections. Thus, persistence does not act as a purely pointwise post-processing filter but as an alarm-state rule with memory.

The SCADA results for turbine T07 corroborate these trends under real operating conditions while also emphasizing their case-dependent nature. In this dataset, all techniques maintain high precision but exhibit uniformly low recall, reflecting the gradual progression and weak observability of the main bearing fault in the available SCADA variables. Conservative indicators, such as the RMWPCA Q-statistic, fail to trigger detections altogether, whereas RMWPCA-pcw and AE provide intermittent but physically interpretable anomaly evidence. As in the simulated cases, temporal persistence rapidly suppresses false alarms but maintains or increases FNs and detection delays when fault signatures remain intermittent, with no persistence range yielding a stable performance plateau. This underscores the difficulty of balancing alarm stability and sensitivity in real SCADA data affected by slow degradation dynamics.

In practical monitoring systems, this observability also depends on sensor placement and on the physical coupling between the monitored variables and the failure mechanism. Sensors weakly coupled to the fault may produce intermittent or low-amplitude health-indicator deviations, making stricter persistence rules more likely to delay or suppress alarm confirmation.

These results demonstrate that temporal persistence is not merely a noise-filtering mechanism but a dominant alarm-confirmation parameter that effectively prioritizes operating regimes in which fault effects are physically observable and temporally sustained. While small persistence values provide substantial gains in alarm reliability at low cost, overly conservative persistence shifts the main operational burden from false alarms to excessive detection delays or missed detections, particularly for incipient or slowly evolving faults. Consequently, alarm outcomes are often governed more strongly by the chosen alarm-confirmation policy than by the anomaly detection algorithm itself.

From a practical standpoint, persistence tuning should therefore be treated as a practical alarm-configuration problem, explicitly accounting for fault severity, degradation dynamics, and operating context, rather than as a fixed heuristic embedded within detection architectures. Because the implemented persistence rule governs both alarm activation and return to the normal state, its configuration affects not only the first detection time but also the duration and stability of alarm states. In operational settings, persistence need not be static and may be adjusted as systems transition across different operating campaigns, load profiles, or maintenance phases. Framing persistence as a configurable alarm-confirmation policy provides a practical basis for configuring anomaly detection systems that balance early warning, alarm stability, and missed-detection control under operational uncertainty.

## 5. Conclusions

This work investigated anomaly detection for wind turbine condition monitoring from a decision-oriented perspective, explicitly separating anomaly indication from alarm confirmation. Using a unified evaluation pipeline, four representative detection families, i.e., IF, OC-SVM, RMWPCA (pcw and Q indicators), and AE, were assessed on controlled OpenFAST simulations of rotor unbalance and on a real SCADA dataset from turbine T07 with a documented main bearing fault. Temporal persistence was treated as an explicit alarm-confirmation parameter, enabling a systematic analysis of how alarm outcomes emerge from the interaction between model-generated evidence and alarm-confirmation policies.

In the simulated scenarios, aggregate metrics obtained under immediate decision rules (n=1) suggested high precision and stable accuracy for most techniques. Time-resolved analyses, however, revealed that many false alarms originated from isolated threshold exceedances occurring before fault onset or during operating-regime transitions, rather than from sustained abnormal behavior. Introducing temporal persistence fundamentally altered this behavior: modest persistence values were sufficient to suppress false alarms almost entirely across all methods, demonstrating that persistence acts as an efficient and largely model-agnostic mechanism for stabilizing alarm states.

At the same time, increasing persistence progressively shifted the dominant error mode toward missed detections and delayed alarms, particularly for low-severity faults. This trade-off was primarily driven by the physical structure of the system rather than by algorithmic limitations. Under low-load operating conditions, fault-induced deviations remained weak and intermittent, preventing anomaly indicators from activating or maintaining persistent alarm states under conservative confirmation rules. As operating conditions became more demanding, fault signatures intensified and became temporally coherent, enabling earlier detection even at higher persistence levels. The evolution of the F1-score confirmed this balance, peaking at low-to-moderate persistence and tending to degrade as persistence became overly conservative.

The SCADA results for turbine T07 corroborated these trends under real operational variability and highlighted their case-dependent nature. All techniques maintained high precision but exhibited low recall, consistent with gradual bearing degradation and limited observability in the available SCADA variables. Persistence again proved decisive: false alarms were rapidly suppressed, but missed detections and detection delays remained high or increased, with no persistence range yielding uniformly stable performance.

The results indicate that alarm behavior in wind turbine anomaly detection is often governed more strongly by the alarm-confirmation policy than by the detection algorithm itself. Persistence should therefore be treated as a configurable alarm-confirmation policy, explicitly balancing false alarms, missed detections, and detection latency while accounting for fault observability, sensor configuration, and data temporal resolution, rather than as a fixed heuristic embedded within detection architectures. Because the implemented rule governs both alarm activation and return to the normal state, its configuration also affects the duration and stability of alarm states. From a practical standpoint, persistence may be adjusted dynamically as operating conditions, load profiles, or maintenance strategies evolve.

These findings are necessarily case-dependent, as they involve a limited set of fault mechanisms and operating conditions. This limitation is particularly relevant for the real-world evaluation, which relies on a single documented industrial SCADA case involving turbine T07 and a main bearing fault. Therefore, the SCADA results should be interpreted as an industrial demonstration of the proposed decision-oriented framework rather than as a statistically exhaustive validation across turbine fleets or fault modes.

Future work should proceed along four specific directions. First, additional industrial SCADA cases involving different turbines, fault modes, and sensor-to-fault coupling conditions should be analyzed to test whether the observed persistence effects remain consistent across broader observability scenarios. Second, more tolerant temporal validation rules, such as *m*-out-of-*n* sliding-window confirmation, should be compared against the consecutive flip-flop rule adopted in this study using the same false-alarm, missed-detection, and detection-latency metrics. Third, simulated scenarios with gradual fault progression should be introduced to complement the discrete severity levels considered here and to evaluate persistence behavior under smoother degradation trajectories. Fourth, fault criticality, expected damage, and cost-based decision criteria should be incorporated so that persistence tuning can be linked to maintenance consequences rather than only to classification metrics. Nonetheless, the study delivers a clear methodological message: reliable and operationally useful alarms in wind turbine monitoring emerge from the joint design of anomaly indicators and alarm-confirmation policies, with temporal persistence playing a central and explicit role.

## Figures and Tables

**Table 1:** Features selected for the simulated unbalance analysis.

Variable ID	Description
NcIMUTVxs_vib_rms	RMS nacelle vibration, axial (m/s)
NcIMUTVxs_vib_0pk	Zero-to-peak nacelle vibration, axial (m/s)
NcIMUTVxs_vib_fft_1x_pk	1× rotor speed amplitude, axial vibration (m/s)
NcIMUTVys_vib_rms	RMS nacelle vibration, vertical radial (m/s)
NcIMUTVys_vib_0pk	Zero-to-peak nacelle vibration, vertical radial (m/s)
NcIMUTVys_vib_fft_1x_pk	1× rotor speed amplitude, vertical radial vibration (m/s)
NcIMUTVzs_vib_rms	RMS nacelle vibration, horizontal radial (m/s)
NcIMUTVzs_vib_0pk	Zero-to-peak nacelle vibration, horizontal radial (m/s)
NcIMUTVzs_vib_fft_1x_pk	1× rotor speed amplitude, horizontal radial vibration (m/s)
WindHubVelX_mean	Mean wind speed (m/s)
GenSpeed_mean	Mean generator/high-speed shaft angular speed (rpm)
RotTorq_mean	Mean shaft torque (kN·m)
RotPwr_mean	Mean rotor power (kW)
RotSpeed_mean_rpm	Mean rotor angular speed (rpm)
GenPwr_mean	Mean generated power (kW)
GenTq_mean	Mean generator torque (kN·m)

**Table 2:** Selected SCADA variables for turbine T07 (main bearing fault).

Variable ID	Description
AmbWindSpeed	Ambient wind speed (m/s)
GenBearTemp	Generator bearing 1 temperature (°C)
GenBear2Temp	Generator bearing 2 (drive-end) temperature (°C)
GrdProdPwr	Grid production power (kW)

**Table 3:** Final configuration of anomaly detection models for simulated cases.

Technique	Final Configuration	Performance Reference
IF	contamination = 0.02; $n_{estimators}$ = 256; ${\mathrm{Max}}_{Samples}$ = 1; ${\mathrm{Max}}_{Features}$ = 1	ACC train: 97.96% (no noise), 97.96% (10% noise); ACC test: 98.72% (no noise), 98.86% (10% noise)
OC-SVM	One-class, RBF kernel; γ = 0.03125; ν = 0.01; 20 support vectors	ACC train: 98.22% (no noise), 98.22% (10% noise); ACC test: 98.31% (no noise), 98.55% (10% noise)
RMWPCA (pcw; Q)	w = 12; no centering; second central moment instead of covariance; confidence level (pcws; Q) = 99.973%	-
AE	9 hidden layers; architecture = [16, 13, 8, 5, 3, 2, 3, 5, 8, 13, 16]; activation = *tanh*; L1 = 0; L2 = 0; confidence level (MSE) = 99.973%	-

**Table 4:** Final configuration of anomaly detection models for turbine T07 case.

Technique	Final Configuration	Performance Reference
IF	contamination = 0.07; $n_{estimators}$ = 512; ${\mathrm{Max}}_{Samples}$ = 1; ${\mathrm{Max}}_{Features}$ = 1	ACC train: 98.00%; ACC test: 98.16%
OC-SVM	One-class, RBF kernel; γ = 0.03125; ν = 0.01; 20 support vectors	ACC train: 99.00%; ACC test: 99.12%
RMWPCA (pcw; Q)	w = 480; no centering; second central moment instead of covariance; confidence level (pcws; Q) = 99.973%	-
AE	3 hidden layers; architecture = [4, 2, 1, 2, 4]; activation = *tanh*; L1 = 0; L2 = 0; confidence level (MSE) = 99.973%	-

**Table 5:** Raw model outputs and the corresponding health indicator definitions.

Technique	Raw Model Output	HI Definition Used in This Work	HI Type
IF	anomaly score s(x)	$HI\left(t\right)=s\left(x_{t}\right)$ (scaled)	Implicit (model-native), scalar
OC-SVM	decision function f(x)	HI(t)= $-f(x_{t})$ (distance to boundary)	Implicit (model-native), scalar
RMWPCA (pcw; Q)	Q(t); ${\mathrm{pcw}}_{i}\left(t\right)$	$HI_{Q(t)}=Q\left(t\right)$;	Explicit (derived), vector
		$HI_{\mathrm{pcw}(t)}$ = $\left[{\mathrm{pcw}}_{1}\left(t\right),{\mathrm{pcw}}_{2}\left(t\right),{\mathrm{pcw}}_{3}\left(t\right)\right]$	
AE	reconstruction error	HI(t) = MSE[x(t),$\hat{x}\left(t\right)$]	Explicit (derived), scalar

**Table 6:** Performance metrics (Accuracy, Precision, Recall, F1-score) and confusion-matrix counts (TPs, TNs, FPs, FNs) across all techniques and simulated conditions.

Scenario	Technique	Accuracy	Precision	Recall	F1-Score	TPs	TNs	FPs	FNs
no noise; 3× baseline unbalance	IF	0.76	0.89	0.60	0.72	300	462	38	200
	OC-SVM	0.81	0.98	0.64	0.77	318	492	8	182
	RMWPCA-pcw	0.67	0.99	0.35	0.52	175	499	1	325
	RMWPCA-Q	0.56	0.98	0.12	0.21	60	499	1	440
	AE	0.83	0.99	0.66	0.80	332	498	2	168
10% RMS noise; 3× baseline unbalance	IF	0.77	0.89	0.62	0.73	308	461	39	192
	OC-SVM	0.81	0.98	0.63	0.76	314	492	8	186
	RMWPCA-pcw	0.73	0.97	0.47	0.63	236	492	8	264
	RMWPCA-Q	0.53	1.00	0.07	0.12	33	500	0	467
	AE	0.82	0.99	0.65	0.78	323	496	4	177
no noise; 5× baseline unbalance	IF	0.76	0.89	0.60	0.72	300	462	38	200
	OC-SVM	0.81	0.98	0.64	0.77	318	492	8	182
	RMWPCA-pcw	0.83	1.00	0.67	0.80	335	499	1	165
	RMWPCA-Q	0.78	1.00	0.55	0.71	277	499	1	223
	AE	0.93	1.00	0.85	0.92	427	498	2	73
10% RMS noise; 5× baseline unbalance	IF	0.76	0.89	0.60	0.72	300	462	38	200
	OC-SVM	0.81	0.98	0.64	0.77	318	492	8	182
	RMWPCA-pcw	0.83	0.98	0.67	0.80	337	492	8	163
	RMWPCA-Q	0.69	1.00	0.38	0.55	191	500	0	309
	AE	0.90	0.99	0.80	0.89	402	496	4	98

**Table 7:** Performance metrics (Accuracy, Precision, Recall, F1-score) and confusion-matrix counts (TPs, TNs, FPs, FNs) for turbine T07.

Scenario	Technique	Accuracy	Precision	Recall	F1-Score	TPs	TNs	FPs	FNs
Turbine T07 (main bearing fault)	IF	0.61	0.89	0.26	0.41	1214	4276	143	3418
	OC-SVM	0.59	0.93	0.22	0.35	998	4339	80	3634
	RMWPCA-pcw	0.63	0.99	0.27	0.42	1246	4412	7	3386
	RMWPCA-Q	0.49	-	0.00	-	0	4419	0	4632
	AE	0.55	0.93	0.12	0.21	555	4379	40	4077

## Data Availability

The original contributions presented in this study are included in the article. Further inquiries can be directed to the corresponding author.

## Abbreviations

The following abbreviations are used in this manuscript:

AE	Autoencoder
FNs	False Negatives
FPs	False Positives
HI	Health Indicator
$index_{det}$	detection index
IF	Isolation Forest
OC-SVM	One Class Support Vector Machine
MSE	Mean Squared Error
pcw	percentage component weight
RMWPCA	Referenced Moving Window Principal Component Analysis
$t_{fault}$	Fault onset time
TNs	True Negatives
TPs	True Positives

## References

[1] Wang Q., Wang X., Li R. (2025). Energy transition and environmental sustainability: The interplay with natural resource rents and trade openness. Humanit. Soc. Sci. Commun..

[2] Katekawa M.E. (2023). Safety and risk assessment in wind energy: Analysis of fire accidents. Brazil Windpower 2023.

[3] Pérez J.M.P., Márquez F.P.G., Tobias A., Papaelias M. (2013). Wind turbine reliability analysis. Renew. Sustain. Energy Rev..

[4] Elsahhar A.U., Ezzat A.A., Elsabbagh A., Elbanhawy A.Y. (2025). Analysis of failure and maintenance records in aging wind farms to inform end-of-life asset management. Wind Energy.

[5] de Souza G.F.M., Caminada Netto A., de Melani A.H.A., de Michalski M.A.C., da Silva R.F. (2021). Reliability Analysis and Asset Management of Engineering Systems.

[6] Yeter B., Garbatov Y., Guedes Soares C. (2020). Risk-based maintenance planning of offshore wind turbine farms. Reliab. Eng. Syst. Saf..

[7] Tidriri K., Braydi A., Kazmi H. (2021). Data-driven decision-making methodology for prognostic and health management of wind turbines. Proceedings of the 2021 Australian & New Zealand Control Conference (ANZCC).

[8] Zio E. (2022). Prognostics and health management (PHM): Where are we and where do we (need to) go in theory and practice. Reliab. Eng. Syst. Saf..

[9] de Custodio L.D.R.S., de Melani A.H.A., Nogueira W.F., Zachariadis D.C., de Souza G.F.M. (2025). Synthetic monitoring data generation for fault detection in wind turbines. Proceedings of the 35th European Safety and Reliability Conference and the 33rd Society for Risk Analysis Europe Conference.

[10] Dabetwar S., Ekwaro-Osire S., Dias J.P., Hubner G.R., Franchi C.M., Pinheiro H. (2023). Mass imbalance diagnostics in wind turbines using deep learning with data augmentation. ASCE-ASME J. Risk Uncertain. Eng. Syst. Part B Mech. Eng..

[11] Schmidt J.O., Aires L.F., Hubner G.R., Pinheiro H., Gamarra D.F.T. (2024). LSTM neural networks using the SMOTE algorithm for wind turbine fault prediction. ASCE-ASME J. Risk Uncertain. Eng. Syst. Part B Mech. Eng..

[12] Bonacina F., Miele E.S., Corsini A. (2022). On the use of artificial intelligence for condition monitoring in horizontal-axis wind turbines. IOP Conf. Ser. Earth Environ. Sci..

[13] Bindingsbø O.T., Singh M., Øvsthus K., Keprate A. (2023). Fault detection of a wind turbine generator bearing using interpretable machine learning. Front. Energy Res..

[14] Chokr B., Chatti N., Charki A., Lemenand T., Hammoud M. (2024). Bi-LSTM autoencoder SCADA based unsupervised anomaly detection in real wind farm data. Proceedings of the 2024 IEEE International Conference on Prognostics and Health Management (ICPHM).

[15] Menezes D., Mendes M., Almeida J.A., Farinha T. (2020). Wind farm and resource datasets: A comprehensive survey and overview. Energies.

[16] Barber S., Izagirre U., Serradilla O., Olaizola J., Zugasti E., Aizpurua J.I., Milani A.E., Sehnke F., Sakagami Y., Henderson C. (2023). Best practice data sharing guidelines for wind turbine fault detection model evaluation. Energies.

[17] Ntafalias A., Weissenfeld A., Visvardi S., Tsakanikas S., Papadopoulos P. (2025). Descriptor: Six-month monitoring dataset from a ten-turbine onshore wind farm in Greece (SMD10TOWFGR). IEEE Data Descr..

[18] McKinnon C., Carroll J., McDonald A., Koukoura S., Infield D., Soraghan C. (2020). Comparison of new anomaly detection technique for wind turbine condition monitoring using gearbox SCADA data. Energies.

[19] McKinnon C., Carroll J., McDonald A., Koukoura S., Plumley C. (2021). Investigation of isolation forest for wind turbine pitch system condition monitoring using SCADA data. Energies.

[20] Yu P., Jia L. (2022). Wind power data cleaning based on autoencoder-isolation forest. Proceedings of the 2022 7th International Conference on Power and Renewable Energy (ICPRE).

[21] Campoverde-Vilela L., Feijóo M.d.C., Vidal Y., Sampietro J., Tutivén C. (2023). Anomaly-based fault detection in wind turbine main bearings. Wind Energy Sci..

[22] Gbashi S.M., Olatunji O.O., Adedeji P.A., Madushele N. (2025). Control chart-integrated machine learning models for incipient fault detection in wind turbine main bearing. Discov. Artif. Intell..

[23] Nogueira W.F., Melani A.H.d.A., Souza G.F.M.d. (2025). Wind turbine fault detection through autoencoder-based neural network and FMSA. Sensors.

[24] Hoffmann M.A., Lasch R. (2025). Unlocking the potential of predictive maintenance for intelligent manufacturing: A case study on potentials, barriers, and critical success factors. Schmalenbach J. Bus. Res..

[25] de Michalski M.A.C., de Souza G.F.M. (2022). Comparing PCA-based fault detection methods for dynamic processes with correlated and non-Gaussian variables. Expert Syst. Appl..

[26] de Melani A.H.A., de Michalski M.A.C., da Silva R.F., de Souza G.F.M. (2021). A framework to automate fault detection and diagnosis based on moving window principal component analysis and Bayesian network. Reliab. Eng. Syst. Saf..

[27] Du S., Wan Y., Zhang C., Zhang S. (2023). Anomaly root cause analysis for wind turbines based on denoising autoencoder and sparse estimation. Proceedings of the 2023 IEEE 12th Data Driven Control and Learning Systems Conference (DDCLS).

[28] Latiffianti E., Sheng S., Ding Y. (2022). Wind turbine gearbox failure detection through cumulative sum of multivariate time series data. Front. Energy Res..

[29] Venkatasubramanian V., Rengaswamy R., Kavuri S.N. (2003). A review of process fault detection and diagnosis. Part II: Qualitative models and search strategies. Comput. Chem. Eng..

[30] Venkatasubramanian V., Rengaswamy R., Yin K., Kavuri S.N. (2003). A review of process fault detection and diagnosis. Part I: Quantitative model-based methods. Comput. Chem. Eng..

[31] Venkatasubramanian V., Rengaswamy R., Yin K., Kavuri S.N. (2003). A review of fault detection and diagnosis. Part III: Process history based methods. Comput. Chem. Eng..

[32] Liu F.T., Ting K.M., Zhou Z.-H. (2008). Isolation forest. Proceedings of the 2008 Eighth IEEE International Conference on Data Mining.

[33] Aljanaideh K.F., Al Saaideh M., Zhang L., Al Janaideh M. (2025). Fault detection and localization of wind turbine sensors using output-only measurements. IEEE Sens. J..

[34] Schölkopf B., Platt J.C., Shawe-Taylor J., Smola A.J., Williamson R.C. (2001). Estimating the support of a high-dimensional distribution. Neural Comput..

[35] Santos P., Villa L., Reñones A., Bustillo A., Maudes J. (2015). An SVM-based solution for fault detection in wind turbines. Sensors.

[36] Michalski M.A.C., Melani A.H.A., da Silva R.F., Souza G.F.M., Hamaji F.H. (2021). Fault detection and diagnosis based on unsupervised machine learning methods: A Kaplan turbine case study. Energies.

[37] Bank D., Koenigstein N., Giryes R. (2021). Autoencoders. arXiv.

[38] Michelucci U. (2022). An introduction to autoencoders. arXiv.

[39] Rumelhart D.E., Hinton G.E., Williams R.J. (1988). Learning internal representations by error propagation. Readings in Cognitive Science.

[40] Hao C., Wang K., Li Y., Yang B., Miao Q. (2019). A data enlargement strategy for fault classification through a convolutional auto-encoder. MATEC Web Conf..

[41] da Silva R.F., de Melani A.H.A., de Michalski M.A.C., de Souza G.F.M. (2022). Failure mode and observability analysis (FMOA): An FMEA-based method to support fault detection and diagnosis. Book of Extended Abstracts for the 32nd European Safety and Reliability Conference.

[42] Wan G., He B., Schweitzer H. (2024). The art of centering without centering for robust principal component analysis. Data Min. Knowl. Discov..

[43] Cadima J., Jolliffe I.T. (2009). On relationships between uncentred and column-centred principal component analysis. Pak. J. Stat..

[44] Chen S., Guo W. (2023). Auto-encoders in deep learning: A review with new perspectives. Mathematics.

[45] Huang L., Pan X., Liu Y., Gong L. (2023). An unsupervised machine learning approach for monitoring data fusion and health indicator construction. Sensors.

[46] Lei Y., Li N., Guo L., Li N., Yan T., Lin J. (2018). Machinery health prognostics: A systematic review from data acquisition to RUL prediction. Mech. Syst. Signal Process..

[47] Jackson J.E., Morris R.H. (1957). An application of multivariate quality control to photographic processing. J. Am. Stat. Assoc..

[48] Jackson J.E., Mudholkar G.S. (1979). Control procedures for residuals associated with principal component analysis. Technometrics.

[49] Tang Z., Shi X., Zou H., Zhu Y., Yang Y., Zhang Y., He J. (2023). Fault diagnosis of wind turbine generators based on stacking integration algorithm and adaptive threshold. Sensors.

